# Supplementary material for: Ingenol-3-Angelate Enhances the B Cell Inhibitory Potential of Mesenchymal Stem Cells, Leading to Marked Alleviation of Lupus Symptoms in MRL.faslpr Mice
Source: Int J Mol Sci. 2024 Nov 25;25(23):12625. doi: 10.3390/ijms252312625 (PMC11641090; doi:10.3390/ijms252312625)
Supplement: Supplementary file 1 [file ijms-25-12625-s001.zip › ijms-3274564-supplementary.pdf]

Supplementary Figure

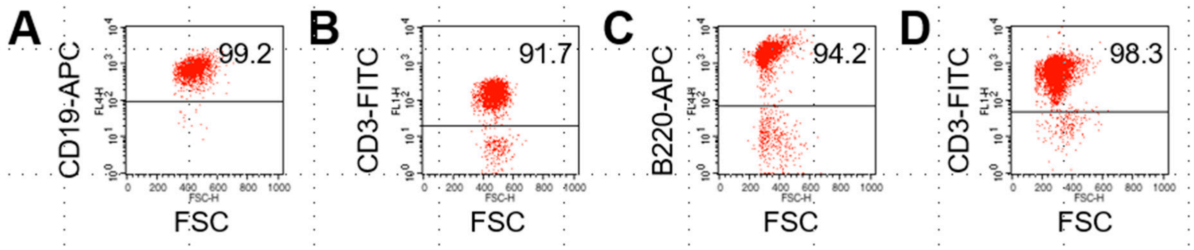

Figure S1. Purity of isolated B and T cells. Human B cells **(A)** and T cells **(B)** were isolated from human PBMCs, while mouse B cells **(C)** and T cells **(D)** were isolated from the spleens of MRL.*Fas*^{lpr} mice. Purity was assessed by flow cytometry. Human cells were stained with an APC-conjugated antibody against human CD19 or an FITC-conjugated CD3 antibody (BD Biosciences). Mouse cells were stained with an APC-conjugated antibody against mouse B220 or an FITC-conjugated CD3 antibody (BD Biosciences).

Supplementary Table

Table S1. Sequences of primers used for RT-qPCR

COX-2	sense, 5′- CCCTTGGGTGTCAAAGGTAA-3′
	antisense, 5′- GCCCTCGCTTATGATCTGTC-3′
FasL	sense, 5′-CTGTGTGCATCTGGCTGGTAGA-3′
	antisense, 5′-GGTTCTGGTTGCCTTGGTAGGA-3″
IDO1	sense, 5′-CCATATTGATGAAGAAGTGGGCT-3′
	antisense, 5′-GATCAGGCAGATGTTTAGCAATGA-3′
iNOS	sense, 5′-ACGTGCGTTACTCCACCAAC-3′
	antisense, 5′-CATAGCGGATGAGCTGAGCA-3′
IL-1	sense, 5′-ACAGATGAAGTGCTCCTTCC-3′
	antisense, 5′-GTCGGAGATTCGTAGCTGGA-3′
IL-6	sense, 5′- AGACAGCCACTCACCTCTTCAG-3′
	antisense, 5′- TTCTGCCAGTGCCTCTTTGCTG-3′
IL-10	sense, 5′- TCTCCGAGATGCCTTCAGCAGA-3′
	antisense,5′-TCAGACAAGGCTTGGCAACCCA-3′
PD-L1	sense, 5′- TGCAGCCAGGTCTAATTGTTTT-3′
	antisense, 5′-TGGCATTTGCTGAACGCATTT-3′
TGF-	sense, 5′-AATTGAGGGCTTTCGCCTTAG-3′
	antisense, 5′-CCGGTAGTGAACCCGTTGAT-3′
-actin	sense, 5′- CATTAAGGAGAAGCTGTGCT-3′
	antisense, 5′- GTTGAAGGTAGTTTCGTGGA-3′

Supplementary Materials

Original images of Western blots and microscopy. When the signals of experimental samples were strong, we briefly exposed blots to the ECL solution in the case of that sample.

Several size markers were not visible in original images, since blots were relatively weakly exposed to the ECL solution.

For the figures, the size marker was loaded onto the gel alongside the samples during the Western blot procedure. However, only the strongly exposed bands of the size marker were visible due to the strong exposure of the ECL solution (Ex Fig. 3 p38, p-p38). The experimental process was carried out accurately, and the position of the size marker was visually confirmed during the experiment.
